# A Curative Immune Profile One Week after Treatment of Indian Kala-Azar Patients Predicts Success with a Short-Course Liposomal Amphotericin B Therapy

**DOI:** 10.1371/journal.pntd.0000764

**Published:** 2010-07-27

**Authors:** Smriti Mondal, Pradyot Bhattacharya, Mehebubar Rahaman, Nahid Ali, Rama Prosad Goswami

**Affiliations:** 1 Infectious Diseases and Immunology Division, Indian Institute of Chemical Biology, Kolkata, India; 2 Department of Tropical Medicine, School of Tropical Medicine, Kolkata, India; New York University School of Medicine, United States of America

## Abstract

**Background:**

The present pilot study investigating the minimum dose for short-course single and double-dose treatment of kala-azar with an apparently new liposomal formulation of amphotericin B, Fungisome, led to identification of immunological components for early detection of success and/or failure to cure.

**Methods:**

Patients were treated with 5, 7.5 (single-dose) and 10 mg/kg body weight (5 mg/kg double-dose) of Fungisome. Immunological investigations involving plasma cytokines and antigen-specific lymphoproliferation and cytokine responses from PBMCs were carried out before, 1 week after Fungisome treatment, at the time of relapse, and again after conventional amphotericin B treatment.

**Results:**

At 1-month follow-up all the patients showed 100% initial cure. However, total doses of 5, 7.5 and 10 mg/kg Fungisome showed 60%, 50% and 90% cure, respectively, at 6-months posttreatment. Patients successfully cured demonstrated downregulation of IL-12 and IL-10 in plasma, and two-fold or more elevation of IFN-γ, IL-12 and TNF, and significant down-regulation of IL-10 and TGF-β in culture supernatants 1-week posttreatment irrespective of drug-dose. A differential immune profile, involving insignificant decline in IL-10 and IL-12 in plasma and negligible elevation of IFN-γ, IL-12 and TNF, and persistence of IL-10, despite decline in TGF-β in culture supernatants, in apparently cured individuals, corresponded with relapse within 6-months of treatment.

**Conclusion:**

Immunological investigations revealed significant curative and non-curative immunomodulation 1-week posttreatment, correlating with successful cure and relapse, respectively. Although immune-correlation was dose-independent, almost consistent curative response in patients treated with the highest dose 10 mg/kg reflected a definitive impact of the higher-dose on the immune response.

**Trial registration name and number:**

Clinical Trials Registry - India (CTRI) CTRI/2009/091/000764

## Author Summary

Visceral leishmaniasis (VL) is a potentially fatal disease without treatment, characterized by prolonged fever, enlargement of spleen and liver, anaemia and weight loss. Treatment for VL is difficult, as it requires prolonged and painful application of toxic drugs with adverse side effects. It is therefore important to develop alternative satisfactory therapies for VL. Herein, we report the efficacy of a new liposomal formulation of amphotericin-B, Fungisome, and the immunological changes that take place 1-week after treatment. Patients treated with 5 and 7.5 mg/kg (single-dose) and 10 mg/kg (5 mg/kg double-dose) of Fungisome showed 60%, 50% and 90% successful cure at 6-month posttreatment, respectively. Successfully cured patients showed reduced IL-12 and IL-10 levels in the plasma and two-fold or more increase in Th1 type-cytokines IFN-γ, IL-12 and TNF, and down-regulation of immunosuppressive factors IL-10 and TGF-β in the culture supernatants, 1-week after treatment independent of drug-dose. Insignificant decrease of plasma IL-12 and IL-10, negligible increase of Th1-cytokines, and persistence of IL-10, despite decrease in TGF-β in culture supernatants, correlated with relapse within 6-months of treatment. These interesting results pave the way for further testing of this drug as a new alternative in the chemotherapy of leishmaniasis.

## Introduction

Visceral leishmaniasis (VL) or kala-azar, caused by members of *Leishmania donovani* complex, is the most severe form of leishmaniasis involving uncontrolled parasitization of liver, spleen and bone marrow. If left untreated VL is generally fatal [1]. Although the disease is endemic in 62 countries, 90% of the estimated 500,000 new cases occur in the rural areas of India, Nepal, Bangladesh, Sudan and Brazil [2]. Pentavalent antimonial is the standard therapy in the endemic regions. However, in north Bihar, India, 34–65% of the VL patients are now refractory to this treatment [3], [4].

Amphotericin B (AmB) at 15–20 mg/kg body weight renders high cure rate of ∼100% in the antimonial unresponsive regions of Bihar [5]. However, serious nephrotoxicity, intravenous administration with constant monitoring, and prolonged hospitalization limit its use [6]. Furthermore, AmB stimulates production of proinflammatory cytokines including TNF-α [7], [8], [9] which although contributes to the antimicrobial activity of AmB also increases its infusion related toxicities (fever, chill, and shivering) [10]. With few satisfactory alternative therapies available, treatment of VL is a challenging task. Liposomal amphotericin B, Ambisome, has the highest therapeutic index of current antileishmanial drugs and reduces the toxicities observed with free AmB, ensuring administration of higher doses leading to short treatment courses with reduced side effects [11]. World Health Organization (WHO) recommends a total dose of 10–21 mg/kg of Ambisome for the treatment of VL [12], [13], [14]. However, even with the preferential pricing for developing countries [15], Ambisome is almost 3-fold the price of conventional AmB. To bring down the cost, recent clinical studies to identify minimum effective total dose showed that a single infusion of 5 or 7.5 mg/kg would only leave a fraction (10–20%) of patients needing further treatment [16], [17], [18]. Under these circumstances, an early prediction of lack of response in this fraction of patients who would require more drug for treatment would be desirable. However, there is no means to predict successful cure or failure of drug treatment in kala-azar.

Active VL is characterized by immune suppression with weak T cell responses [19]. In vivo cytokine profile from the serum and at the mRNA level from bone marrow, lymphoid tissues, and splenic aspirate of patients showed mixed Th1/Th2 cytokine response during active disease [20], [21], [22], [23], [24]. In contrast, studies with leishmanial Ag-stimulated PBMC showed Th2-type bias at active VL with decreased or absent IL-2 and IFN-γ production and upregulation of both IL-10 and TGF-β [25], [26], [27], [28], [29]. With the onset of chemotherapy and cure with sodium antimony gluconate (SAG) and AmB, upregulation of IFN-γ, and IL-12 synchronized with a decline in IL-10 and TGF-β levels [25], [26], [27], [28], [29]. In addition to the direct antileishmanial activity, both AmB and SAG showed in vitro immunomodulatory activities on PBMCs resulting in decline of IL-10, with AmB downregulating TGF-β also [29]. So a short-course therapy with liposomal formulation of AmB that maintains the efficacy of AmB and is non-toxic would be the ideal first line treatment for kala-azar.

A liposomal AmB preparation, developed in India and commercially available as Fungisome, is safe and effective for treatment of VL [2]. As for Ambisome, Fungisome is also an intravenous infusion, and a total dose of 15–21 mg/kg showed an efficacy of 90.9–100% against responsive and unresponsive cases of VL [30]. To initiate short-course therapy, we investigated whether a relatively higher dose at a single- (5, and 7.5 mg/kg) or a double-dose (5 mg/kg) (total 10 mg/kg) of Fungisome could be safe and effective for therapy of kala-azar. In addition, we were interested to investigate whether a similar modulation of cytokine profile as observed with AmB treatment could be obtained with Fungisome as early as 1 week after treatment to identify if possible immunological components for early detection of successful treatment and/or failure to cure.

## Materials and Methods

### Subjects

This short-course pilot study was performed between 2006 and 2008 at the School of Tropical 

Medicine (Kolkata, India). Patients were mainly from endemic regions of eastern India diagnosed with active VL. Based on the inclusion exclusion criteria (Protocol S1) patients of all ages and both sexes were potentially eligible if they presented the clinical symptoms of prolonged fever, hepatosplenomegaly, and were confirmed to be VL by K39 strip test and detection of *Leishmania* parasites in the splenic or bone marrow aspirate.

### Study Design

Three groups of VL patients were classified based on their mode of treatment. Group A (n = 10) patients were categorized for treatment (Lifecare Innovations, India) with 5 mg/kg body weight Fungisome single-dose. Group B (n = 10) patients were treated with Fungisome 7.5 mg/kg single-dose, and Group C (n = 10) were treated with Fungisome 5 mg/kg×2 doses on subsequent days. 100 ml Fungisome was dissolved in 100 ml of normal saline and infused i.v. over 2 hours. Patients were monitored during infusion and further for 24 hours for any adverse effects, and were discharged 1-week after treatment. Some of the patients who suffered relapse within 6-months of treatment were further treated with conventional AmB (Sarabhai Piramal Pharmaceuticals, India) (total dose 20 mg/kg body weight) by i.v. drip in dextrose solution on alternate days. Longitudinal heparinized blood samples from the patients were collected at different time points. Biochemical and hematological characteristics are shown in Table S1.

### Ethics review board approval and informed consent

The study was approved by the Institutional Review Board and Ethical Committee, Calcutta School of Tropical Medicine, Kolkata. Written informed consent was obtained from each patient enrolled in the study. A copy of the patient consent form was submitted to the Ethical Committee.

### Leishmanial antigen (LAg) preparation

Leishmanial antigen (LAg) was prepared from promastigotes of *L. donovani*

(MHOM/IN/1983/AG83) as described earlier [31]. Briefly, stationary phase promastigotes harvested after the third or fourth passages were washed four times in ice-cold 20 mM phosphate-buffered saline (PBS, pH 7.2) and suspended in 5 mM cold Tris-HCl buffer (pH 7.6). The suspension was vortexed six times for 2 min each with a 10-min interval on ice and then centrifuged at 2310×*g* for 10 min. The crude ghost membrane pellet obtained was resuspended in the same buffer and sonicated on ice three times for 1 min in an ultrasonicator. The suspension was centrifuged at 5190×*g* for 30 min and the supernatant containing the LAg was harvested and stored at −70°C. The amount of protein obtained from 1-g cell pellet was ∼14 mg, as assayed by Lowry *et al* [32].

### Proliferation assay

Heparinized blood samples for isolation of PBMCs from VL patients, and healthy volunteers (n = 5) from the Indian Institute of Chemical Biology (IICB), Kolkata, India (non endemic for VL), were obtained after written informed consent and was approved by The Ethical Committee on Human Subjects, IICB. Briefly, PBMCs isolated from the blood samples by density sedimentation on Histopaque-1077 (Sigma-Aldrich) (400×g, 30 min at RT), were washed and resuspended in RPMI 1640 supplemented with 10% FCS (Sigma, St Louis, MO), 2 mM L-glutamine, penicillin (100 U/ml), and streptomycin (100 µg/ml). PBMCs (1×10^6^/ml) of VL patients were cultured in triplicates in 96-well flat-bottom tissue culture plates (Nunc) and stimulated with and without LAg (12.5 µg/ml) for 4 days at 37°C in 95% humidified air with 5% CO_2_. 1 µCi of [^3^H] Thymidine (sp. act. 5 Ci/mM; Amersham Biosciences) was added to the wells and cultured for another 18–24 hours. Thymidine uptake was measured in a β scintillation counter (Beckman Instruments, Fullerton, CA).

### Cytokine analysis

Plasma from VL patients were collected at different time points and stored at −20°C for cytokine analysis. PBMCs of these patients were cultured in the presence of LAg and media as described above. After 96 hours supernatants were collected for cytokine analysis. To study the direct effect of AmB and Fungisome to modulate cytokine production, PBMCs (1×10^6^/ml) from healthy controls were incubated with various non-toxic concentrations of AmB (0, 0.031, 0.062, 0.125, 0.25, 0.5 µg/ml) and Fungisome (0, 0.031, 0.062, 0.125, 0.25, 0.5, 1 µg/ml) with or without LPS (1 µg/ml) for 48 hours at 37°C in 95% humidified air with 5% CO_2_. Culture supernatants were collected and stored at −70°C until use. IFN-γ, IL-12 (p40), TNF and IL-10 (BD OptEIA ELISA kit; BD Biosciences) were measured according to the manufacturers' instructions. For total TGF-β measurement, culture supernatants were acidified to activate the latent TGF-β by adding 1 N HCl for 10 min and neutralized with 1.2 N NaOH in 0.5 M HEPES. Plasma samples were activated with 2.5 N acetic acid in 10 M urea for 10 min and neutralized with 2.7 N NaOH in 1 M HEPES. TGF-β was captured with monoclonal anti-TGF-β1, MAB240, and detected with biotinylated polyclonal anti-TGF-β1, BAF240 (R&D Systems). The standard curve was prepared using rTGF-β1 (R&D Systems) suspended in culture medium. The color reaction was performed using avidin-HRP and tetramethylbenzidine and read at OD 450 nm.

### Statistical calculation

Statistical analysis was performed using the nonparametric Wilcoxon matched pairs signed rank test for paired samples and the Mann-Whitney U test for unpaired samples. One-way ANOVA followed by Tukey's multiple comparison test was performed to assess the differences among various groups (GraphPad Software, Inc., San Diego, CA.). *P*<0.05 was considered to be significant.

## Results

### Response to therapy

**Figure 1:**
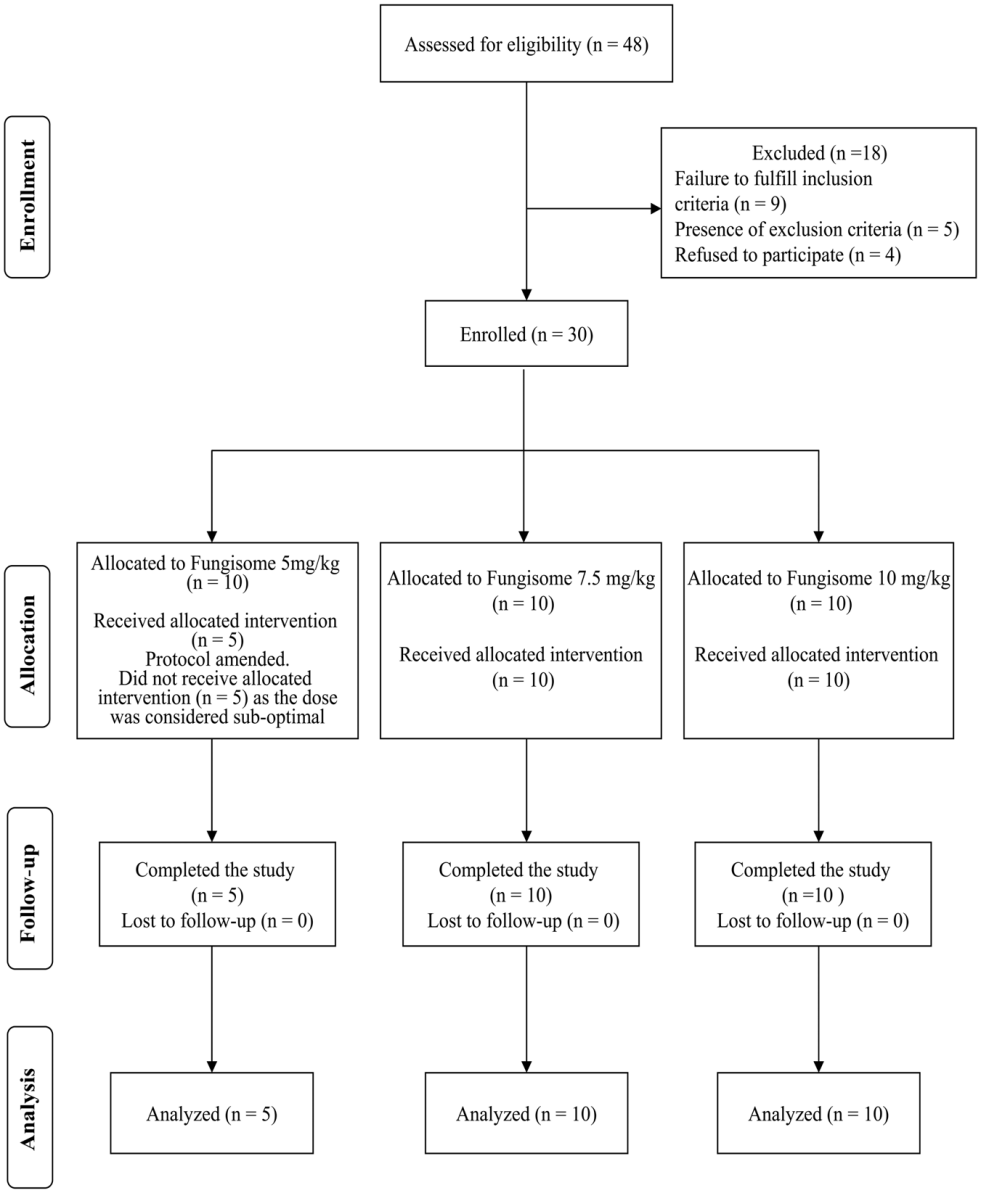
Flowchart summarizing the clinical study.

Three groups of patients were categorized based on the mode of treatment with Fungisome; Group A (5 mg/kg body weight single dose, n = 10), Group B (7.5 mg/kg single dose, n = 10) and Group C (5 mg/kg×2 doses on subsequent days, n = 10). Figure 1 shows the flow of participants through the dose finding study. Within 3 months of study initiation, 2 out of 5 patients (40%) in Group A returned with relapse. So this dose was considered suboptimal and the protocol was amended to continue treatment only in Group B and C. At 1-month follow up, initial response to treatment was noted and all the patients were considered clinically cured. Within 6-months follow-up, 5 out of 10 patients (50%) in Group B and 1 out of 10 patients (10%) in Group C (Table 1) experienced relapse. Therefore cure rate 6-months posttreatment was 60% in Group A, 50% in Group B and 90% in Group C. The other 5 enrolled patients in Group A, who did not receive treatment with Fungisome, and the patients who suffered relapse in all the three groups, were treated with conventional AmB (20 mg/kg) and were cured. All the three doses of Fungisome were remarkably well tolerated (Table S2).

**Table 1:** Responses to Fungisome treatment in Indian kala-azar patients.

Response	Group A(n = 5)	Group B(n = 10)	Group C(n = 10)
Treatment withdrawn	0	0	0
Initial cure at 1 month	5	10	10
Relapse	2	5	1
Lost to follow-up	0	0	0
Definitive cure at 6-month no. (%)	3(60)	5(50)	9(90)

**NOTE**. Data are no. of patients, unless otherwise indicated. Group A received a total dose of 5 mg/kg; Group B, 7.5 mg/kg; Group C, 10 mg/kg of Fungisome.

### Circulating plasma cytokines during active VL and at posttreatment

For immunological studies two groups of patients were categorized, responders who were successfully cured 6-months posttreatment and non-responders who suffered relapse within 6-months of treatment. The mean plasma cytokine levels of both Th1-type, IFN-γ, IL-12, TNF, and Th2 type immunosuppressive cytokines IL-10 and TGF-β were elevated during active disease (Table 2). 1-week after treatment, there was significant downregulation of IL-12 and IL-10 (*P*<0.005) in the responders compared to untreated condition, however, there was no significant change in the levels of IFN-γ, TNF and TGF-β (Table 2). The fall in IL-10 in the responders was also significant (*P*<0.05) when compared with non-responders 1-week posttreatment. Difference in the cytokine levels of non-responders was significant neither at 1-week posttreatment nor at relapse compared to untreated patients. However, after cure with AmB there was significant downregulation again restricted to IL-12 and IL-10 (*P*<0.05) compared to active diseased and relapsed patients (Table 2). Although there was a mixed Th1-Th2 type cytokine profile in the plasma of VL patients during active disease and after treatment with Fungisome and AmB, a significant fall in IL-10 and IL-12 in both corresponded with cure.

**Table 2:** Plasma cytokine profile of Fungisome treated patients.

Cytokines (pg/ml)	Before treatment (n = 21)	1 week after Fungisome treatment		At the time of relapse (n = 8)	After AmB treatment (n = 8)
		Responders (n = 13)	Non-responders (n = 8)		
IFN-γ	23.79 (2.07)	22.22 (1.79)	15.63 (2.65)	23.19 (4.6)	23.35 (2.33)
IL-12	222.8 (19.76)	107.2 (18.14)^a^	172.5 (25.83)	182.9 (27.20)	100.4 (19.93)a, c
TNF	19.44 (7.74)	28.22 (15.11)	17.13 (4.90)	22.36 (7.72)	11.18 (2.80)
IL-10	62.69 (6.01)	30.58 (2.65)a, b	47.69 (6.61)	48.31 (5.22)	28.50 (1.55)a, c
TGF-β	1507 (193.1)	1450 (330.8)	1700 (313.5)	1581 (393.2)	1400 (320.7)

Plasma cytokine levels were measured by ELISA from all the 3 groups of patients, before treatment, 1 week after Fungisome treatment, at the time of relapse, and again after AmB treatment. Data are represented as mean (SEM). *P* values were calculated using Mann-Whitney *U* test for unpaired samples; *P*<0.05 was considered significant.

a Significantly different from VL patients before treatment (*P*<0.005).

b Significantly different from non-responders 1 week after Fungisome treatment (*P*<0.05).

c Significantly different from the time of relapse (*P*<0.05).

### Antigen-specific lymphoproliferation and Th1-type cytokine analysis before and 1-week after Fungisome treatment

Consistent with prior observations [19], [29], VL patients at active disease showed impaired lymphoproliferation in response to LAg-stimulation (Table 3, Figure S1). Remarkably, 1-week after Fungisome treatment there was significant enhancement in lymphoproliferation in the responders of all the three groups of patients (*P*<0.0001) (Table 3) especially in Group B and Group C (*P*<0.05) (Figure S1). However, non-responders showed no or low lymphoproliferation 1-week posttreatment and at the time of relapse. Subsequent treatment with AmB (20 mg/kg) resulted in significantly (*P*<0.05) increased lymphoproliferation.

**Table 3:** LAg-specific lymphoproliferation and cytokine analysis before and after treatment with Fungisome.

Before treatment (n = 21)		1-week after Fungisome treatment		At the time of relapse (n = 8)	After AmB treatment (n = 8)
		Responders (n = 13)	Non responders (n = 8)		
**Lymphoproliferation**					
Stimulation index (SI)	1.20 (0.03)	1.84 (0.12)^a^	1.41 (0.12) b	1.37 (0.12)	2.90 (0.12)^a^
**Cytokines (pg/ml)**					
IFN-γ	15.35 (3.34)	60.48 (20.74)^a^	20.75 (4.67)^b^	46.25 (24.94)	60.13 (18.85)^a^
IL-12	34.46 (6.44)	91.03 (12.96)^a^	44.53 (4.48)^b^	58.09 (21.29)	135.05 (32.95)^a^
TNF	4.52 (1.68)	17.58 (3.42)^a^	4.91 (0.97)^b^	7.06 (3.32)*	19.00 (4.02)^a^
IL-10	58.19 (4.17)	37.86 (9.44)^a^	54.69 (8.31)^b^	52.63 (6.69)	24.22 (4.51)^a^
TGF-β	393.72 (60.75)	222.12 (41.31)^a^	258.1 (43.43)	400.30 (61.47)	208.30 (27.49)^a^

**NOTE**. *Leishmania*-specific cell-mediated immune response in Fungisome treated patients. PBMCs were stimulated with 12.5 µg/ml LAg for 5 days and lymphoproliferation was measured by [^3^H]thymidine incorporation for the last 18–24 hours of culture. Cytokine levels were measured from supernatants of similar cultures after 96 hours calculated over background levels from all the 3 groups, before treatment, 1 week after Fungisome treatment, at the time of relapse and again after AmB treatment. Data are represented as mean (SEM). *P* values were calculated using Mann-Whitney *U* test for unpaired samples; *P*<0.05 was considered significant.

a Significantly different from VL patients before treatment (*P*<0.05).

b Significantly different from responders 1 week after Fungisome treatment (*P*<0.05).

*: Excluding 1 patient in Group A, who had very high level of TNF due to some unknown reason.

LAg-stimulated cytokine production during disease demonstrated low levels of IFN-γ, IL-12 and TNF (Table 3) measured over background levels. 1 week after Fungisome treatment there were two or more fold increase in antigen-stimulated IFN-γ production in the responders of all the groups (*P*<0.05) (Table 3), more consistent in Group C (Figure S1) when compared with untreated patients and non-responders (Table 3). In contrast, there was no such increase in patients who suffered relapse 1-week posttreatment. Although there was significant upregulation of IL-12 and TNF in the responders of all the groups who were cured 6-months posttreatment compared to the untreated (*P*≤0.0006) and non-responders (*P*≤0.005) (Table 3) there was low or insignificant increase in these cytokines in non-responders following treatment. Separate analysis indicated that immunological responses were dose-independent (Figure S1). Therefore, two or more fold upregulation of IFN-γ, IL-12 and TNF, 1 week after Fungisome treatment, could predict successful cure at 6-months posttreatment irrespective of the drug dose. At the time of relapse the patients had moderate levels of LAg-specific IFN-γ and IL-12 and low levels of TNF (Table 3), which increased significantly (*P*<0.05) after completion of AmB treatment.

### Antigen-specific immunosuppressive cytokine analysis before and 1-week after treatment with Fungisome

Though IL-10 and TGF-β are not considered typical Th2 cytokines both have been implicated as immunosuppressive factors in human VL. Prior to treatment there were high levels of LAg-specific IL-10 and TGF-β, calculated over background levels, in most of the patients of all the three groups (Table 3, Figure S2). There was significant (*P*<0.005) downregulation of IL-10 in the responders of all the three groups 1-week after Fungisome treatment compared to before treatment (Table 3) and non-responders (*P*<0.05). However, there was no significant downregulation of IL-10 in the non-responders who suffered relapse. Similarly, TGF-β levels were high prior to treatment in all the patients. 1 week following treatment there was decline in TGF-β in both responders and non responders, although the fall was significant (*P*<0.005) only in the responders of all the groups. Persisting levels of IL-10 and TGF-β at the time of relapse showed significant decline following treatment with AmB.

### *In vitro* immunomodulatory effects of Fungisome and AmB on human PBMCs

**Figure 2:**
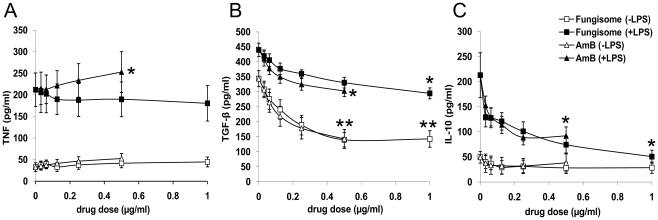
Ability of Fungisome and AmB to modulate TNF, TGF-β and IL-10 production of human PBMCs. PBMCs (1×10^6^) from healthy controls were incubated with various concentrations of Fungisome and AmB with or without LPS (1 µg/ml) for 48 h at 37°C in 95% humidified air with 5% CO_2_. Cytokines (A) TNF, (B) TGF-β and (C) IL-10 were measured by ELISA from supernatants. Each symbol represents mean cytokine level (±SEM) at each dose of the drugs (n = 5). Values for *p* were calculated by ordinary one-way ANOVA followed by Tukey's multiple comparison tests; *P*<0.05 was considered significant. *, *P*<0.02–0.05; **, *P*<0.01.

To compare the in vitro immunomodulatory effects of Fungisome with conventional AmB [9], PBMCs from healthy individuals were cultured for 48 hours in the presence or absence of LPS and various nontoxic concentrations of these drugs (Figure 2). Although AmB significantly (*P*<0.05) upregulated LPS-stimulated TNF production, Fungisome did not increase the TNF level (Figure 2A). Unstimulated PBMCs led to the production of high levels of TGF-β that was enhanced further in the presence of LPS. Both AmB and Fungisome were instrumental in significantly (*P*<0.01) downregulating inherent as well as LPS-induced TGF-β production at concentrations 0.25 µg/ml and above (Figure 2B) and LPS-stimulated IL-10 production (*P*<0.05) in a dose-dependent manner (Figure 2C). The effect was more significant (*P*<0.05) for Fungisome as the highest dose of the drug could be extended to 1 µg/ml.

## Discussion

The present pilot study evaluated the efficacy for short course single- (5, and 7.5 mg/kg) and double-dose (5×2 mg/kg) Fungisome treatment of kala-azar patients. Interestingly, a differential immune profile of the patients with modulation of in vivo and antigen-stimulated cytokine responses was observed before and 1 week after drug treatment which correlated with successful cure or relapse 6-months posttreatment.

Commercially available lipid formulations of AmB have largely remedied the drawbacks of conventional AmB enabling safe and short-duration administration of higher doses for cost-effective treatment. A cure rate of 90.9–100% was reported for Fungisome at 3 mg/kg for 5–7 days (total dose 14–21 mg/kg) [30]. To establish the lower limit of efficacy and reduce treatment duration we initiated Fungisome treatment at 5 and 7.5 mg/kg single-dose, and 5 mg/kg double-doses (total 10 mg/kg) on consecutive days. All the three doses of Fungisome showed an initial clinical cure of 100% at 1-month follow-up. However, total doses of 5, 7.5 and 10 mg/kg of Fungisome, showed 60%, 50% and 90% cure rate at 6-months posttreatment. There are few reports on comparative short-course dose-finding studies with liposomal AmB and these show varying results [33], [34], [35], [36], warranting further testing of single and double-dose regimens of liposomal AmB.

It is well established that infection with *L*. *donovani* and its control are modulated by a range of 

T cell responses and cytokine network. We therefore investigated the immune environment of the patients at 1-week posttherapy. The results interestingly throw light on the possible mechanisms of successful cure and relapse, despite an apparent cure. Investigation of plasma cytokines revealed a mixed Th1–Th2 type profile in the VL patients during active disease as observed earlier [23], [24], [37], [38]. Interestingly, 1-week following treatment resulted in a significant fall in the levels of IL-10 and IL-12 corresponding with cure. Similar fall was also observed in the cured individuals treated with conventional AmB. High serum levels of IFN-γ during active VL do not reconcile with large parasite burdens observed in this disease. Lack of IFN-γ activity may be related to the simultaneous presence of elevated levels of IL-10 and TGF-β, the macrophage deactivating cytokines in human leishmaniasis [39]. The observed fall in IL-10 levels 1-week following treatment is coincident to the control of parasite growth lending support to an important role of this cytokine in human VL.

Investigations on the antigen-specific cytokine production by PBMCs showed an enhanced Th1-type response with upregulated IFN-γ, IL-12 and TNF and reduced IL-10 and TGF-β production 1 week after treatment in VL patients who were cured at 6-months posttreatment irrespective of the drug-dose. IL-12 helps in the expansion of IFN-γ that synergistically acts with TNF-α to activate macrophages to kill *Leishmania* parasites through release of NO [40], [41]. IL-10 and TGF-β neutralize the effects of IFN-γ [37], [39], [42] and favor survival of *Leishmania* by inhibiting NO production by the macrophages [43] thus helping in disease progression [39], [44], [45], [46], [47], [48]. Patients who suffered relapse, showed neither significant elevation of Th1 cytokines nor downregulation of IL-10, at this time point, although TGF-β level was reduced. This differential immune response 1 week posttreatment between the responder and non-responder patients further substantiates the significance of upregulation of Th1 cytokines and simultaneous downregulation of IL-10 for the success of therapy. Further, our studies show that this polarization occurs as early as 1 week of treatment and thus has the potential to serve as markers to predict therapeutic success. Elevation of antigen-specific IL-10 and TGF-β along with the Th1 cytokines at the time of relapse substantiates the significance of immunosuppressive activities of IL-10 and TGF-β. Conventional AmB treatment (20 mg/kg) of relapsed patients generated a dominant Th1-type response as observed earlier [29].

Since cure is a combinatorial effect of drug and immune status of the host in VL, the rationale approach towards antileishmanial chemotherapy would be to potentiate the immune functioning of the host [49] in addition to parasite killing. Targeting of Th1 cell mechanism increased the efficacy of AmB and permitted lower doses to be used with comparable activities [50], [51]. In addition, our previous study revealed that AmB can downregulate IL-10 and TGF-β in the LPS-stimulated PBMCs of healthy individuals [29]. Interestingly, in the present study similar results were maintained by Fungisome. Simultaneous downregulation of IL-10 and TGF-β is important as their combined in vivo blockade led to an apparent sterile immunity in mice infected with *Leishmania* parasites [48]. TNF, responsible for infusion-related toxicities of AmB (10) was increased only in the presence of AmB. No such increase was observed with Fungisome corroborating earlier reports [52] of reduced TNF production by lipid carriers of AmB.

The mechanism of the observed immunoenhancement in Fungisome treated patients was due to the combined effect of the drug's (AmB) parasite killing effect together with unexpectedly rapid regulation of cytokine responses. A significant downregulation of IL-12 and IL-10 in the plasma, elevation of IFN-γ, IL-12 and TNF, and downregulation of IL-10 and TGF-β in PBMCs, in patients' 1-week after treatment corresponded with successful cure irrespective of drug dose. Negligible decline in plasma IL-12 and IL-10 levels, negligible elevation of IFN-γ, IL-12 and TNF and persistence of IL-10, despite decline in TGF-β in culture supernatants of apparently cured individuals, predicted disease relapse within 6-months posttreatment. Although the immune modulation was dose-independent, low cure rates in Group A and B corresponded with fall in TGF-β levels without much effect on IL-10 expression in some of the patients who eventually relapsed. In contrast, treatment with the highest dose in Group C simultaneously downregulated IL-10 and TGF-β, and upregulated IFN-γ, IL-12 and TNF. This dual effect might be responsible for the successful cure, with only one relapse, in this Group. Fungisome maintained increased immunomodulatory activity of AmB at doses that are toxic with the conventional AmB. To extend this clinically appealing, novel approach, studies should be initiated with higher total doses of 10 mg/kg (5 mg/kg double-dose) and 15 mg/kg (7.5 mg/kg double-dose) of Fungisome with larger number of patients to better define the role of this new liposomal AmB.

## Supporting Information

Protocol S1 Detailed study protocol.
(0.04 MB DOC)

Table S1 Clinical and biochemical characteristics of VL patients at baseline and after Fungisome treatment.
(0.04 MB DOC)

Table S2 Adverse reactions during Fungisome treatment.
(0.02 MB DOC)

Figure S1 Leishmania specific T cell proliferation, IFN-γ and IL-12 response in Fungisome treated VL patients. Longitudinal samples of PBMCs were isolated from Fungisome treated patients before the onset of treatment, 1 week after chemotherapy, at the time of relapse and again after the completion of AmB therapy. Three different groups were distinguished on the basis of the drug dose. Group A (n = 5) patients were treated with a single dose of 5 mg/kg and Group B (n = 8) with 7.5 mg/kg of Fungisome, whereas Group C (n = 8) were treated with 10 mg/kg (5 mg/kg double dose) of Fungisome. Cells were stimulated with 12.5 µg/ml of LAg for 5 days and lymphoproliferation was measured by [3H] thymidine incorporation for the last 18–24 hours of culture. Proliferation is indicated as stimulation index (SI) for each patient. Cytokines, IFN-γ and IL-12 were measured by ELISA from the supernatants of similar cultures for 4 days. Each symbol represents an individual patient. Values for p were calculated using Wilcoxon matched pairs signed rank test for paired samples; P<0.05 were considered significant.
(7.08 MB TIF)

Figure S2 Leishmania specific TNF, IL-10 and TGF-β levels before and after Fungisome treatment in VL patients. Longitudinal samples of PBMCs were isolated from Fungisome treated patients before the onset of treatment, 1 week after chemotherapy, at the time of relapse and again after the completion of AmB therapy. Three different groups were distinguished on the basis of the drug dose. Group A (n = 5) were treated with a single dose of 5 mg/kg and Group B (n = 8) with 7.5 mg/kg of Fungisome, whereas Group C (n = 8) were treated with 10 mg/kg (5 mg/kg ´ 2) of Fungisome. TNF, IL-10 and TGF-β were measured by ELISA from the supernatants of cultures stimulated with LAg (12.5 µg/ml) for 4 days. Each symbol represents an individual patient. Values for p were calculated using Wilcoxon matched pairs signed rank test for paired samples; P<0.05 were considered significant. *, excluding one patient in Group C with very high levels of IL-10 due to some unknown reason.
(11.61 MB TIF)

Checklist S1 CONSORT checklist.
(0.06 MB DOC)
