# Supplementary material for: A Curative Immune Profile One Week after Treatment of Indian Kala-Azar Patients Predicts Success with a Short-Course Liposomal Amphotericin B Therapy
Source: PLoS Negl Trop Dis. 2010 Jul 27;4(7):e764. doi: 10.1371/journal.pntd.0000764 (PMC2910702; doi:10.1371/journal.pntd.0000764)
Supplement: Protocol S1 — Detailed study protocol. (0.04 MB DOC) [file pntd.0000764.s001.doc]

**Detailed Study Protocol**

**Subjects**

This open-label dose finding short-course therapy pilot study was carried out between June 2006 and January 2008 at the School of Tropical Medicine (STM) (Kolkata, India) associated with kala-azar treatment in eastern India. Follow-up at 6-month posttreatment continued to January 2008. Patients mainly from endemic regions of eastern India, Bihar and West Bengal, diagnosed with active VL were admitted to STM for treatment. All the diagnostic and treatment procedures were performed in STM.

**Sample Design**

VL patients were confirmed if they presented consistent clinical symptoms of prolonged fever, hepatosplenomegaly and were confirmed to be VL by K39 strip test and detection of *Leishmania* amastigotes in the splenic or bone marrow aspirate. They were further screened and recruited for the study using the following inclusion exclusion criteria.

Inclusion criteria:

1. Patients of all ages and both sexes
2. Consistent signs and symptoms of active VL.
3. Confirmed diagnosis of VL with positive identification of parasite from bone marrow or splenic aspirate or K39 strip test.
4. Confirmed VL patients fully informed about the risk of treatment who provide written informed consent (self or parent or authorized relative) of their willingness to undergo treatment with the new drug (Fungisome) at the specified dose.
5. Patients willing to participate in all treatment and follow-up visits regularly at monthly interval for six months at the out-patient department at STM.

Exclusion criteria:

1. HIV-positive individuals with VL
2. VL patients already receiving antileishmanial drugs will be excluded from the study.
3. Patients who have hypersensitivity to the drug or its constituents.
4. Patients who have associated disease altering liver function tests.
5. Pregnant women.

Withdrawal from study

If the patient is non compliant and does not come for follow-up upto six months, he/she will be withdrawn from the study.

Baseline and follow-up assessments

Detail history and clinical examination of the patients were carried out including ECG, chest X-ray, ultrasonography of whole abdomen, urine R/E, M/E, C/S. Blood sampling was done for examination of peripheral blood and limited biochemical analysis (liver function tests, serum urea and creatinine, serum Na K, fasting and postprandial blood sugar) and screening for HIV. These routine tests were carried out for all the study participants before therapy and during monthly follow-up visit upto six months posttreatment.

**Sample size**

Depending on the availability of Fungisome as donated by Lifecare Innovations, India, we aimed to enroll 10 patients in each treatment arm of this dose finding study. A total of 48 eligible patients were screened; 14 were excluded by the preceding inclusion exclusion criteria and 4 elected not to participate and 30 patients were finally enrolled (Fig. 1).

**Study Design**

The study design was an open-label, randomized, parallel group, multiple arm pilot study where confirmed VL patients who fulfilled the inclusion exclusion criteria were enrolled for treatment with Fungisome (Fungisome, Lifecare Innovations, India). An independent statistician prepared randomization envelops by the use of a computer generated random number table. The sealed envelops were then distributed to the enrolled subjects, to randomly assign them to one of the following total dose groups; 5 mg/kg single shot (n = 10), 7.5 mg/kg single shot (n = 10) and 5 mg/kg double shot (total 10 mg/kg) (n = 10) Fungisome.

**Study Protocol**

Three groups of randomly selected VL patients were categorized based on their mode of treatment. Group A (n = 10) patients were treated with Fungisome 5 mg/kg bd.wt single dose. Group B (n = 10) patients were treated with Fungisome 7.5 mg/kg bd.wt single dose, and Group C (n = 10) were treated with Fungisome 5 mg/kg bd.wt × 2 doses on subsequent days. Fungisome was sonicated on a bath sonicator for 45 minutes before use. Injectable Fungisome was dissolved in 5% dextrose solution (1:1 vol/vol ratio) and initially 1 mg of the drug was infused intravenously in 5 mins. If no hypersensitive reaction was observed, the rest of the drug was infused over 2-3 hours. Patients were monitored during the infusion and for further 24 hours for any adverse effects and were discharged one week after treatment. Treatment with other potentially anti-leishmanial drugs was forbidden during the study.

Some of the patients who suffered relapse within 6 months of Fungisome treatment were further treated with conventional AmB (Sarabhai Piramal Pharmaceuticals, India) (total dose 20 mg/kg of body weight) by i.v. drip in dextrose solution on alternate days. Longitudinal heparinized blood samples from these three treatment groups (Group A, B and C) were collected before the initiation of treatment with Fungisome and one week after the completion of treatment. Blood samples were also collected at the time of relapse from the patients who suffered relapse within 6- months of Fungisome treatment and after their completion of treatment with AmB. Clinical and biochemical analysis including, spleen size assessment, complete blood cell count, haemoglobin and serum urea creatinine levels, were assessed before treatment with Fungisome and at one month follow up. Infusion

related adverse drug effects were recorded during Fungisome therapy.

**Evaluation of clinical response**

Primary outcome or initial cure at one-month was determined by resolution of fever, reduction of spleen and liver enlargement and improvement in clinical and biochemical parameters as well as performance status. Secondary outcome or final cure assessed after 6 months posttreatment, required the person to be healthy and to have no signs and symptoms of relapse. The recurrence of signs and symptoms of VL (indicated by spleen enlargement, fever, and the detection of parasites in the splenic aspirate smears) denoted relapse.

**Ethics review board approval and informed consent**

The study protocol followed the International Conference on Harmonization (ICH) Good Clinical Practice Guidelines in full compliance with principle of the Declaration of Helsinki. The study was approved by the Institutional Review Board and Ethical Committee, Calcutta School of Tropical Medicine, Kolkata. A copy of the patient consent form was submitted to the Ethical Committee. The physicians explained the nature of the investigation and the risks involved to each patient (or parent or relative in case of minors), prior to recruitment. Written informed consent was obtained from each patient enrolled in the study and the patient/parent was informed that he/she was free to voluntarily withdraw from the study at any time.
