# Supplementary material for: A Curative Immune Profile One Week after Treatment of Indian Kala-Azar Patients Predicts Success with a Short-Course Liposomal Amphotericin B Therapy
Source: PLoS Negl Trop Dis. 2010 Jul 27;4(7):e764. doi: 10.1371/journal.pntd.0000764 (PMC2910702; doi:10.1371/journal.pntd.0000764)
Supplement: Table S1 — Clinical and biochemical characteristics of VL patients at baseline and after Fungisome treatment. (0.04 MB DOC) [file pntd.0000764.s002.doc]

**Table S1**

# Clinical and laboratory characteristics of VL patients at baseline and after treatment with different doses of Fungisome

|  | **Group A (5 mg/kg)**  **n = 5** | | | **Group B (7.5 mg/kg)**  **n = 10** | | | **Group C (10mg /kg)**  **n = 10** | | |
| --- | --- | --- | --- | --- | --- | --- | --- | --- | --- |
| Characteristics | Day 0 | Day 7 | Day 30 | Day 0 | Day 7 | Day 30 | Day 0 | Day 7 | Day 30 |
| Sex  Male  Female | 2  3 |  |  | 8  2 |  |  | 7  3 |  |  |
| Age (yr) | 13.20 ± 3.2 |  |  | 11.95 ± 3.15 |  |  | 19.22 ± 3.6 |  |  |
| Prior antimony therapy, no. (%) of patients | 1(20) |  |  | 1(10) |  |  | 1(10) |  |  |
| Duration of illness (months) | 3.8 ± 0.86 |  |  | 5.15 ± 0.95 |  |  | 5 ± 1 |  |  |
| Body wt (kg) | 24.00 ± 4.39 | 24.2 ± 4.34 | 25.2 ± 4.42 | 23.81 ± 3.98 | 24 ± 4.14 | 25.15 ± 4.22 | 38.70 ± 3.55 | 38.9 ± 3.57 | 41.20 ± 3.53 |
| Karnofski score | 69 ± 1.87 |  | 85 ± 1.58 | 69.5 ± 1.57 |  | 88 ± 1.86 | 71 ± 1 |  | 89 ± 1.45 |
| Spleen size (cm) | 9.7 ± 2.47 | 7.1 ± 2.16 | 6 ± 2.12 | 8.15 ± 0.83 | 6.05 ± 0.50 | 4.55 ± 0.45^b^ | 10.80 ± 0.94 | 6.41 ± 0.37 | 3.45 ± 0.50^a^ |
| Splenic score | 3.80 ± 1.48 |  |  | 4 ± 0.70 |  |  | 3.9 ± 0.46 |  |  |
| Hemoglobin (g/dl) | 7.8 ± 0.33 |  | 8.52 ± 0.46 | 7.20 ± 0.34 |  | 9.30 ± 0.70^a^ | 7.54 ± 0.36 |  | 9.28 ± 0.42^a^ |
| WBC count (cells/µl) | 2800 ± 221 |  | 4300 ± 272 | 3105 ± 530 |  | 4970 ± 660^a^ | 2686 ± 563 |  | 5414 ± 636^b^ |
| Platelet count (x 10^5^ platelets/mm^3^) | 1.16 ± 0.11 |  | 2.08 ± 0.12 | 1.24 ± 0.12 |  | 2.23 ± 0.10^b^ | 1.33 ± 0.14 |  | 2.50 ± 0.15^b^ |
| Serum creatinine level (mg/dl) | 0.76 ± 0.04 |  | 0.85 ± 0.038 | 0.8 ± 0.044 |  | 0.83 ± 0.050 | 0.79 ± 0.056 |  | 0.87 ± 0.074 |
| Blood urea nitrogen (mg/dl) | 19.6 ± 4.74 |  | 25.6 ± 4.70 | 19.8 ± 1.05 |  | 30 ± 2.14 | 19.20 ± 1.36 |  | 25.80 ± 2.37 |

**NOTE**. Data are mean values ± SEM, unless otherwise indicated. Group A received a total dose of 5 mg/kg; Group B, 7.5 mg/kg; Group C, 10 mg/kg of Fungisome. *P* values were calculated using Wilcoxon matched pairs signed rank test for paired samples; *P* < 0.05 were considered significant.

^a^ *P* < 0.005, compared with the day 0 value.

^b^ *P* < 0.01 - 0.05, compared with the day 0 value.
