# Supplementary material for: A Curative Immune Profile One Week after Treatment of Indian Kala-Azar Patients Predicts Success with a Short-Course Liposomal Amphotericin B Therapy
Source: PLoS Negl Trop Dis. 2010 Jul 27;4(7):e764. doi: 10.1371/journal.pntd.0000764 (PMC2910702; doi:10.1371/journal.pntd.0000764)
Supplement: Table S2 — Adverse reactions during Fungisome treatment. (0.02 MB DOC) [file pntd.0000764.s003.doc]

**Table S2**

# Adverse reactions during Fungisome treatment

| Adverse effect | Group A  n = 5 | Group B  n = 10 | Group C  n = 10 |
| --- | --- | --- | --- |
| None | 2 (40) | 4 (40) | 4 (40) |
| Chills | 0 | 1(10) | 3 (30) |
| Fever | 3 (60) | 5 (50) | 5 (50) |
| Backache /Body ache | 1 (20) | 0 | 0 |
| Vomiting | 0 | 2 (20) | 1 (10) |

**NOTE**. Data are no. (%) of patients. Group A received a total dose of 5 mg/kg single dose; Group B, 7.5 mg/kg single dose; Group C, total 10 mg/kg of Fungisome in 5 mg/kg × 2 doses on consecutive days.
